# Identification of cell lines CL-14, CL-40 and CAL-51 as suitable models for SARS-CoV-2 infection studies

**DOI:** 10.1371/journal.pone.0255622

**Published:** 2021-08-02

**Authors:** Claudia Pommerenke, Ulfert Rand, Cord C. Uphoff, Stefan Nagel, Margarete Zaborski, Vivien Hauer, Maren Kaufmann, Corinna Meyer, Sabine A. Denkmann, Peggy Riese, Kathrin Eschke, Yeonsu Kim, Zeljka Macak Safranko, Ivan-Christian Kurolt, Alemka Markotic, Luka Cicin-Sain, Laura Steenpass

**Affiliations:** 1 Department of Bioinformatics and Databases, Leibniz Institute DSMZ - German Collection of Microorganisms and Cell Cultures, Braunschweig, Germany; 2 Department of Vaccinology and Applied Microbiology, Helmholtz Centre for Infection Research, Braunschweig, Germany; 3 Department of Human and Animal Cell Lines, Leibniz Institute DSMZ - German Collection of Microorganisms and Cell Cultures, Braunschweig, Germany; 4 Dr. Fran Mihaljević University Hospital for Infectious Diseases, Zagreb, Croatia; 5 Centre for Individualized Infection Medicine, a Joint Venture of Helmholtz Centre for Infection Research and Medical School Hannover, Hannover, Germany; Cornell University, UNITED STATES

## Abstract

The SARS-CoV-2 pandemic is a major global threat that sparked global research efforts. Pre-clinical and biochemical SARS-CoV-2 studies firstly rely on cell culture experiments where the importance of choosing an appropriate cell culture model is often underestimated. We here present a bottom-up approach to identify suitable permissive cancer cell lines for drug screening and virus research. Human cancer cell lines were screened for the SARS-CoV-2 cellular entry factors *ACE2* and *TMPRSS2* based on RNA-seq data of the Cancer Cell Line Encyclopedia (CCLE). However, experimentally testing permissiveness towards SARS-CoV-2 infection, we found limited correlation between receptor expression and permissiveness. This underlines that permissiveness of cells towards viral infection is determined not only by the presence of entry receptors but is defined by the availability of cellular resources, intrinsic immunity, and apoptosis. Aside from established cell culture infection models CACO-2 and CALU-3, three highly permissive human cell lines, colon cancer cell lines CL-14 and CL-40 and the breast cancer cell line CAL-51 and several low permissive cell lines were identified. Cell lines were characterised in more detail offering a broader choice of non-overexpression in vitro infection models to the scientific community. For some cell lines a truncated *ACE2* mRNA and missense variants in *TMPRSS2* might hint at disturbed host susceptibility towards viral entry.

## Introduction

It is of no debate that the overcoming of the pandemic SARS-CoV-2 pathogen spreading across the continents urgently needs joint efforts in the scientific community. Within a few months in the midst of spring 2020 the pandemic was accompanied with a high case fatality rate of up to 20% for vulnerable risk groups [1] and high excess mortality in Europe monitored by EuroMOMO (https://www.euromomo.eu/graphs-and-maps) [2]. High transmissibility of SARS-CoV-2 with R0 of ∼2.5 exceeds that of SARS-CoV, the 2009 influenza A H1N1 virus and even the 1918 influenza pandemic [3], probably due to its airborne transmission via droplets and aerosol [4–6]. During summer time, the portion of asymptomatic cases in a small Italian village were quantified to 43% [7] and since pre-/asymptomatic virus carriers were observed to be contagious, these are thought to drive the pandemic [8]. Re-infection also has been reported to complicate the pandemic course [9].

While various vaccines are rolled out [10–12], options for antiviral treatment for severely diseased patients are still sparse. Neutralising antibodies are promising drugs [13], while small molecules like remdesivir [14] have not yet proven great benefits [15, 16]. Importantly, all future antivirals, when becoming available and broadly used, face the challenge of SARS-CoV-2 escape mutations [17, 18]. Thus, it is clear that drug screening and basic research in SARS-CoV-2 is and will be of continuous importance.

Current investigations are mainly limited to the VERO-E6 African green monkey cell line, human lung cancer-derived CALU-3 and human colon carcinoma cell line CACO-2. Using non-human cells to study SARS-CoV-2 bears the inherent risk to draw conclusions from findings that result from species-specific cell properties. The anti-SARS-CoV-2 properties of hydroxychloroquine were found using VERO-E6, while later studies detected no effect in human cells and patients [19, 20]. In addition, the virus mutates rapidly when replicating in VERO-E6 [21–23]. Overexpression of host factors, such as TMPRSS2, can overcome individual species-specific artefacts but comes with commonly observed difficulties of stable transgene expression, such as antibiotic selection, non-physiological protein level, or transgene silencing during passaging.

Human cancer cell lines are easily available from public collections, characterised in great detail, and offer a great range of tissue origins, expression patterns, growth rates, and functional properties to choose from. They serve as valuable and valid model systems to study different diseases [24, 25] and have been used specifically for SARS-CoV-2 viral entry [26, 27] or for antiviral studies against this virus [28]. Particularly the two surface proteins ACE2 and TMPRSS2 have been shown to contribute to viral binding and processing [26, 27] and are mainly expressed in bronchial transient secretory cells [29]. Interestingly, high gene expression of *ACE2* is shown for other tissues such as myocardial cells, esophagus epithelial cells, and enterocytes, which might be related to non-respiratory symptoms observed in COVID-19 patients [30–33]. Many virus-host-interaction studies rely on *ACE2/TMPRSS2*-transfected cell lines such as 293T and HELA cells or non-human material such as African green monkey cells VERO-E6 [26, 34, 35]. A recent study validated HPAEpiC as a human AT2 cell-derived pulmonary alveolar epithelial cell line to be susceptible to SARS-CoV-2 [36].

In this study, we have pre-screened and ranked human cancer cell lines of the Leibniz Institute German Collection of Microorganisms and Cell Cultures (DSMZ) for *ACE2* and *TMPRSS2* expression within the rich resource Cancer Cell Line Encyclopedia (CCLE) publicly available RNA-seq data [37]. Selected cell lines were then validated for mRNA and protein levels of ACE2 and TMPRSS2 and tested in SARS-CoV-2 infection experiments, revealing a set of cell lines displaying high and low permissiveness. With these cell lines we devise appropriate, non-overexpressing cell model systems to facilitate experimental SARS-CoV-2 work for the understanding of viral entry, viral replication and to support drug development.

## Results

### Selection of cell model systems

**Fig 1:**
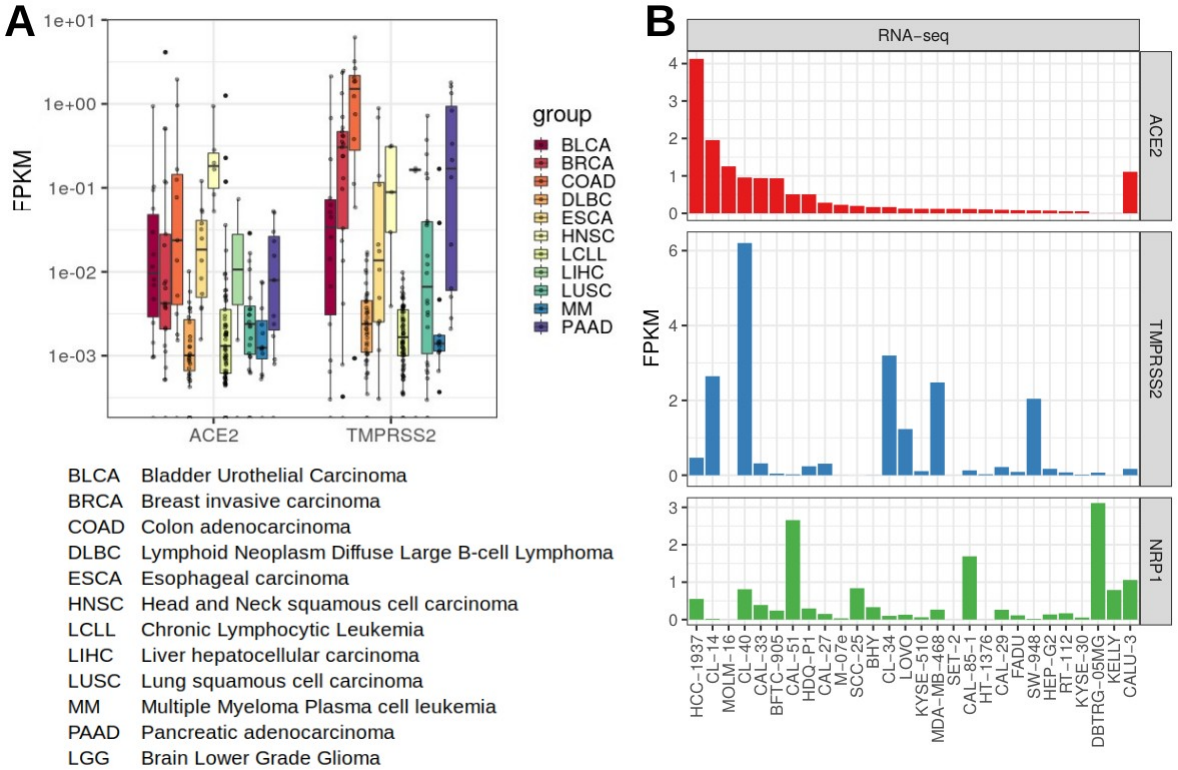
*ACE2* and *TMPRSS2* gene expression data for 301 DSMZ human cell lines in the CCLE RNA-seq data set. A: Expression of *ACE2* and *TMPRSS2* for selected disease entities. RNA-seq data were normalised and calculated to FPKM values. Gene expression for *ACE2* and *TMPRSS2* varies between and within the different tumour species. B: Expression of *ACE2*, *TMPRSS2* and *NRP1*, cell lines sorted to *ACE2* expression levels. Top 25 *ACE2* expressing cell lines were selected for further studies, plus CALU-3 and two further neuronal cell lines with high *NRP1* levels. FPKM—fragments per kilobase million: normalised gene expression data.

Inspired by the publication of Hoffmann et al. pinpointing the host factors ACE2 as SARS-CoV-2 receptor and TMPRSS2 as viral S protein priming serine protease [26], we defined expression of these two factors as pre-selection criteria in our study. Since the Cancer Cell Line Encyclopedia (CCLE) as a comprehensive database provides large-scale sequencing data including mRNA-seq expression data for over 900 cell lines [37], we screened for gene expression of *ACE2* and *TMPRSS2* in this data set, which enabled us to identify potential susceptible cell lines prone to SARS-CoV-2 virus entry. At first, we selected for 301 human cell lines readily in stock at the DSMZ cell lines repository within this data set and visualised gene expression of *ACE2*, which was shown to be the cellular receptor for the viral S protein [38], and *TMPRSS2* known to cleave SARS-CoV S protein [39] (Fig 1A). Gene expression for these two genes differed between and within the various tumour entities. In order to pre-select cell lines to be tested for SARS-CoV-2 permissiveness, the top 25 highest *ACE2* gene expressing cell lines were selected, originating from diverse tumour origins (Fig 1B). Importantly, our short-list included cancer cell lines originating from tissues that are known for SARS-CoV-2 replication in vivo [40], e.g. the tongue squamous cell carcinoma cell lines CAL-27, CAL-33, and the esophageal squamous cell carcinoma cell line KYSE-510. Based on this evidence, we additionally selected the newly established oral squamous cell carcinoma cell lines UPCI-SCC-074 and UPCI-SCC-131, which are not contained in the CCLE dataset. Futhermore, we included the neuronal cells DBTRG-05MG and KELLY with high levels of neuropilin 1 (*NRP1*) mRNA expression (Fig 1B), which has been discussed as receptor for the viral spike protein [41, 42]. *NRP1* expression was most prominent in DBTRG-06MG, CAL-51, CAL-85–1, CALU-3, SCC-25, CL-40, and KELLY (Fig 1B). Since the lung carcinoma cell line CALU-3, the colorectal adenocarcinoma CACO-2, and the green monkey kidney cell line subclones VERO-E6 and VERO-B4 are shown to be permissive for SARS-CoV-2 [26, 27], these were used as positive control cell lines. Altogether we selected 29 human cancer cell lines and four control cell lines.

**Fig 2:**
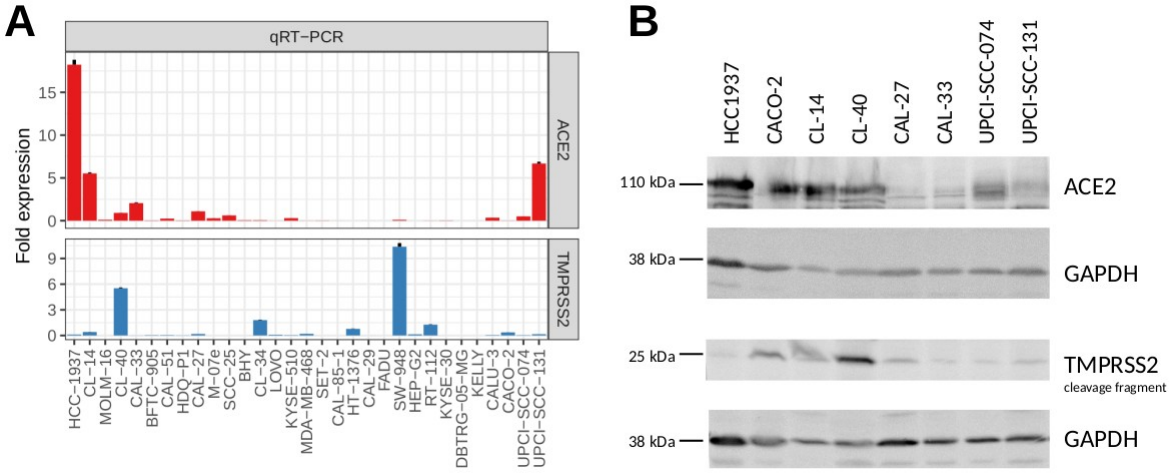
Verified presence of ACE2 and TMPRSS2 in carcinoma cell lines determined by qRT-PCR and western blots. A: Quantification of *ACE2* and *TMPRSS2* transcripts in the selected candidate cell lines by qRT-PCR (normalised to CAL-27 and CL-40). B: Western blot analysis was performed to assess the expression of proteins associated with SARS-CoV-2 cell entry.

In order to validate *ACE2* and *TMPRSS2* expression levels, we performed qRT-PCR on the short-listed cell lines. All selected cell lines exhibited considerable *ACE2* mRNA expression according to the RNA-seq data and/or qRT-PCR except for DBTRG-05MG and KELLY, whereas *TMPRSS2* mRNA levels were particularly higher in CL-40, CL-34, CL-14, and SW-948 (Figs 1B and 2A). Apart from qualitative verification for mRNA expression via RNA-seq data and qRT-PCR (Fig 2A), protein expression of ACE2 and TMPRSS2 was confirmed via western blot for specific cell lines (Fig 2B, S1 Fig).

For expanding our cell line search spectrum, we interrogated the LL-100 cell line data set, comprising RNA-seq and whole-exome sequencing (WES) data of 100 human leukemia and lymphoma cell lines [24]. However, none of these cell lines showed considerable expression of *ACE2* and *TMPRSS2*. This is reflected in the CCLE results, in which cell lines of the hematopoetic lineages exhibited lower gene expression of both genes compared to other cell lines of different origin (see Fig 1A DLBC, LCLL and MM tumour entities).

The presence of the entry factors ACE2 and TMPRSS2 in cancer cell lines of various origins is in accordance with their expression in corresponding primary tissue, where *ACE2* and *TMPRSS2* were detected in cells from multiple tissues including respiratory tract, esophagus and colon [31, 33, 43–45] and virus entry was evidenced in gastro-intestinal tissues [46], organs of the respiratory tract and various other tissues [40].

### Testing for SARS-CoV-2 infectivity and permissiveness

Receptor ACE2 and surface proteinase TMPRSS2 have been shown to play a key role for SARS-CoV-2 cellular entry, however, whether their co-expression is sufficient to allow entry and whether alternative receptors may exist, is still under debate [45]. In order to shed light on infectivity and permissiveness of the appointed cell lines in context of their ACE2/TMPRSS2 expression, we subjected cells to experimental infection with wildtype SARS-CoV-2. Permissiveness to viral infection is defined as the ability of a given host cell to permit the entire viral replication cycle and to eventually release mature virions. Thus, we sampled the supernatant of the short-listed cell lines at four consecutive days following infection with a low amount of inoculum to sensitively detect viral replication.

**Fig 3:**
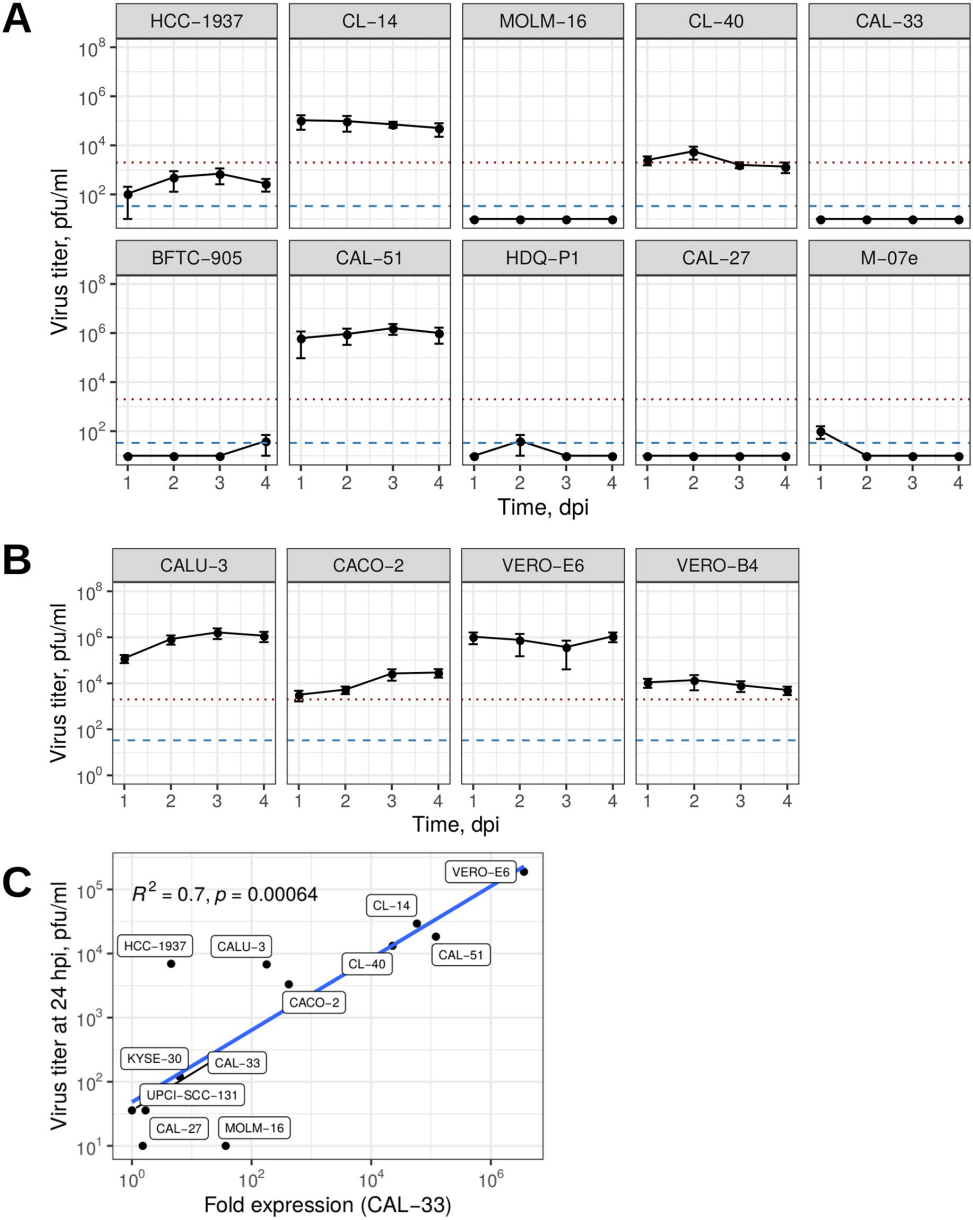
Susceptibility of tumour cell lines toward SARS-CoV-2 infection. Virus titers in the supernatant of infected cell lines at 1–4 days postinfection (dpi) determined by plaque assay for the top 10 ACE2 expressing cell lines (A) and controls (B). The red dotted line indicates the initial inoculum (2000 pfu/ml); the blue dashed line specifies the minimal limit of detection LOD 33.3 pfu/ml. Number of replicates varied between 3–8. pfu: plaque-forming units; mean values with SEM. (C) Predominant productive virus production in highly permissive cell lines seen in the correlation between qRT-PCR measured viral nucleoprotein N mRNA and releasing virions tested by viral plaques. Eight replicates served for the means of the viral titers and one representative expression fold change of two biological replicates qRT-PCR with three technical replicates. Note the logarithmic scaling for both x and y-axis.

Within our short-listed panel, we identified large variation in the degree of permissiveness and virus replication kinetics. Surprisingly, the majority of tested cell lines (15 of 29) showed no detectable virus replication despite the presence of ACE2 and TMPRSS2 (Fig 3A, S2 Fig). Further 11 cell lines showed detectable SARS-CoV-2 infectious titres that did not exceed the amount of the inoculum. Only three cell lines were categorized highly permissive, amplifying the initial virus inoculum by factors of ∼2 to ∼500 between 1 and 4 dpi. These were the colon carcinoma cell lines CL-14 and CL-40, as well as breast carcinoma cell line CAL-51. Infectious titres of SARS-CoV-2 grown on CL-14 and on CAL-51 were concurrent with the widely used CALU-3 (lung carcinoma), CACO-2 (colon carcinoma), VERO-E6 and VERO-B4 (normal African green monkey kidney cells) (Fig 3B). Whereas CL-14 already has been described to be permissive in a preprint [47], the breast carcinoma cell line CAL-51 with high levels of *NRP1* beside *ACE2* and *TMPRSS2* expression and the colon carcinoma cell line CL-40 have not been identified before. Despite comparable peak titres, virus replication kinetics differed largely between cell lines, where CAL-51 and CACO-2 showed increase in titres throughout day three postinfection while VERO-E6, VERO-B4, and CL-14 produced highest levels within 24h, while the viral titer for CL-40 remained close to the level of the initial inoculum of SARS-CoV-2 (Fig 3, Table 1).

**Table 1:** Permissive cell line characteristics.

cell line	Species	Sex	Tissue	Peak viral load, dpi	Maximal peak, pfu/ml	Doubling time
CL-14	human	male	colon	1	1.1 x 10^5^	∼1w
CL-40	human	female	colon	2	5.8 x 10^3^	∼3–4d
CAL-51	human	female	breast	3	1.6 x 10^6^	∼30h*
CALU-3	human	male	lung	3	1.6 x 10^6^	∼84h*
CACO-2	human	male	colon	4	3.0 x 10^5^	80h
VERO-B4	monkey	female	kidney	2	1.4 x 10^5^	∼25h
VERO-E6	monkey	female	kidney	4	1.1 x 10^6^	22h^+^

Peak viral load and maximal peak were determined during this study.

Species, sex, tissue and doubling time were taken from https://www.dsmz.de/collection/catalogue/human-and-animal-cell-lines/catalogue.

*own observation;

^+^ATCC.

Therapeutic neutralising antibodies bind to the viral Spike protein and prevent the interaction with ACE2 [13]. However, it is unclear whether different levels of ACE2 expression on the surface of cells would interfere with the ability to block virus entry. Thus, we used three different permissive cell lines of our panel (CL-14, CAL-51, and VERO-E6) with varying ACE2 levels and performed in vitro neutralisation assays using the therapeutic anti-Spike antibody COR-101 currently used in clinical trials (see S3 Fig) [48]. While the comparability among these cell lines is limited due to several variables (cell density, virus growth kinetic, cell medium, etc.), we observed ≥93% inhibition of virus entry at 100 ng/ml in all three cell lines, compared to the lowest nAb concentration. The inhibition curves suggest a reduced neutralisation efficiency in the CAL-51 breast cancer cell line, which might be attributed to NRP-1-mediated or NRP-1-assisted virus entry [41, 42]. NRP-1 is an alternative or assisting SARS-CoV-2 entry factor that shows elevated levels in CAL-51 cells (see Fig 1A). However, the complete virus neutralisation at sufficient antibody levels suggests either the absence of ACE2-independent entry or the additional inhibition of a Spike/NRP-1 interaction.

In contrast, low-permissive cell lines, such as the breast cancer HCC-1937, the colon adenocarcinoma CL-34, and esophageal squamous cell carcinoma KYSE-30 cell lines showed slow replication kinetics, which was also true for the oral squamous cell carcinoma cell lines UPCI-SCC-074 and UPCI-SCC-131 but not for the neuronal cells DBTRG-05MG and KELLY despite high levels of *NRP1* (Fig 3A, S2 Fig). Non-permissive cell lines showed no detectable SARS-CoV-2 titres at any time after infection.

In order to test for abortive viral cycles particularly in the non-productive virus releasing cell lines, we compared mRNA levels of the nucleoprotein in each three control, high-permissive, low-permissive, and non-permissive cell lines to viral plaques after 24 hpi (Fig 3C). We found a high correlation (*R*^2^ = 0.7) between viral expression and infective virus titers in the tested cell lines, probably reduced by high variation in viral infection of the cell lines at detection limit. Hence, these data argue for the absence of abortive virus replication or defects in releasing mature virions in the low-permissive cell lines.

### Genomic analyses of *ACE2* and *TMPRSS2*

**Fig 4:**
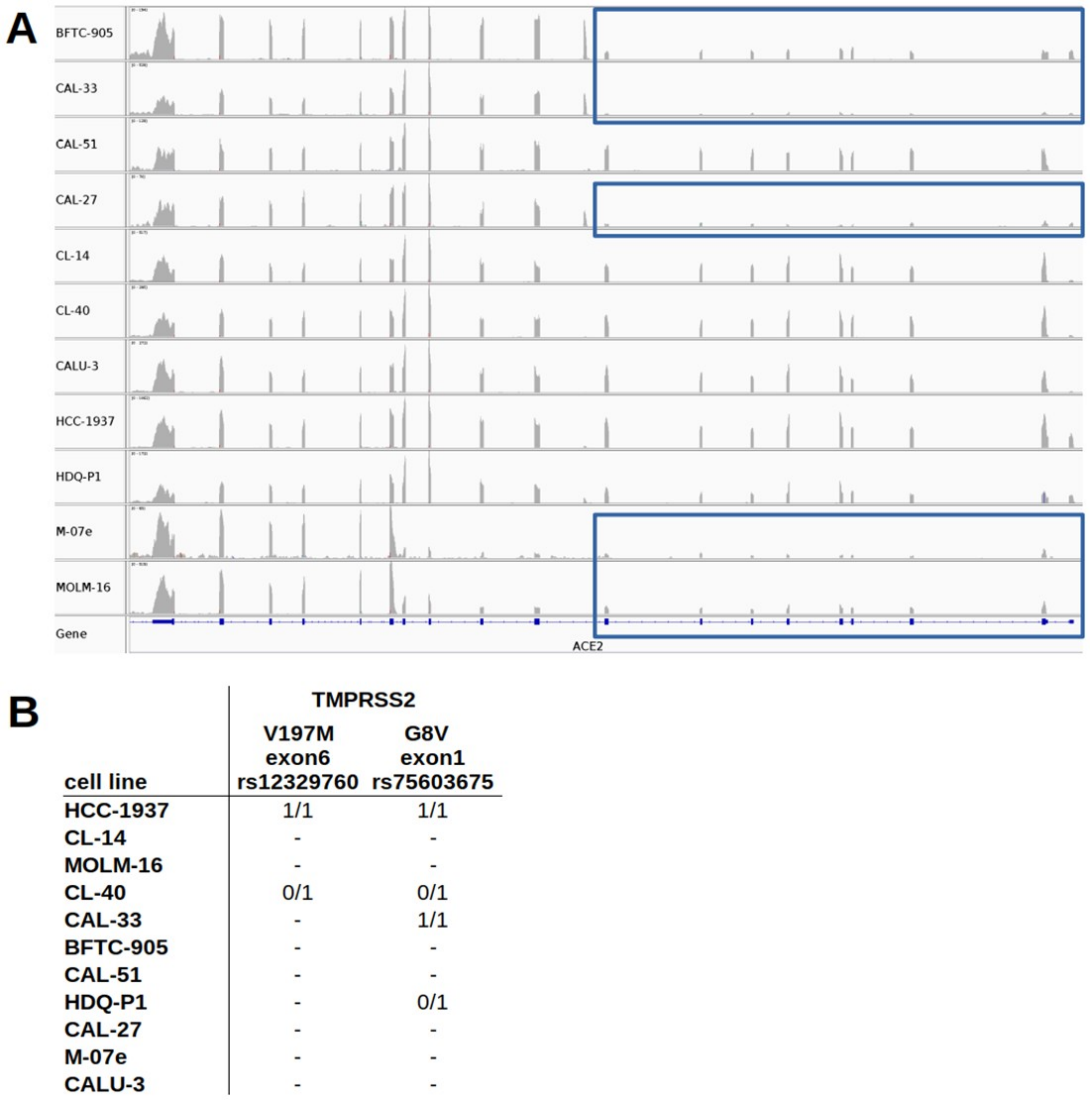
*ACE2* and *TMPRSS2* transcript variants for the cell lines. A: Different exon usage of *ACE2* for the top 10 *ACE2* expressing cell lines plus CALU-3 (see S2 Fig for the top 25 cells). BFTC-905, CAL-33, CAL-27, M-07e, and MOLM-16 show reduced exon expression from exon 1–9 (blue rectangles). Cell lines are ordered alphabetically. *ACE2* is reversely oriented. B: Missense variants in the coding regions of *TMPRSS2* (V197M = rs12329760, G6V = rs75603675) for the selected cell lines. No mutations were found in the coding regions of *ACE2*. 0/1 denotes heterozygous and 1/1 homozygous mutations.

Since we found limited concordance between *ACE2/TMPRSS2* expression and virus permissiveness of the cell lines, we seeked for genomic differences in the receptors. Of note, gene expression of different splice variants of the *ACE2* mRNA became evident comparing the read numbers of the exons as seen in the CCLE RNA-seq data for MOLM-16, CAL-33, BFTC-905, and CAL-27 (Fig 4A, S4 Fig, S1 Table). The truncated *ACE2* lacking exon 1–9 has been reported recently [49, 50].

Furthermore, missense variants were detected in *TMPRSS2* for HCC-1937 (homozygous), CL-40 (heterozygous), CAL-33 (homozygous), and HDQ-P1 (heterozygous) (Fig 4B), namely V197M (V160M) in exon 6 (rs12329760) and/or G8V in exon 1 (rs75603675). We assume that these variations may have a considerable effect on the virus-host interaction.

## Discussion

In this study, we have systematically identified and characterised SARS-CoV-2 permissive cancer cell lines that (except one) have not been published as such before. Of 29 selected human cell lines three tested high-level positive permissive for SARS-CoV-2. These selected cell lines are available from the German repository DSMZ. Cancer cell lines have been an integral tool for basic research and drug screening for decades and still are the preferred choice for the vast majority of virus research labs. For SARS-CoV-2 infection experiments, different replication kinetics as well as different degrees of permissiveness can aid answering specific questions and allow different experimental setups, e.g. in compound screening assays. We found permissiveness to SARS-CoV-2 in only 14 out of 29 cell lines pre-selected for the expression of the known entry factor *ACE2* (Figs 1B and 2). Three of them replicated the virus to high titres (CL-14, CL-40, and CAL-51) and further 11 showed low levels with mainly slow virus kinetics compared to commonly used VERO-E6, CALU-3, and CACO-2 cells (Fig 3, S2 Fig, Table 1).

In order to understand the non-permissiveness of the majority of top 25 *ACE2* expressing cell lines, we closely examined splice and single nucleotide variants. *ACE2* splice variants at the N-terminus, containing eight amino acids critical for binding to the receptor binding domain (RBD) of the spike protein of SARS-CoV-2, are predicted to disturb SARS-CoV-2 attachment [51]. One splice variant lacking exon1-exon9 was observed in e.g. MOLM-16, CAL-33, and CAL-27 (Fig 4A, S4 Fig, S1 Table), cell lines that indeed were not shown to be non-permissive despite their pronounced *ACE2* gene expression (Fig 1B). This truncated *ACE2* isoform has been reported to be interferon inducible [49] and fails to bind to SARS-CoV-2 [50].

Whereas the lacking permissiveness for SARS-CoV-2 of the above mentioned cell lines is likely to be explained by a truncated *ACE2* transcript (Fig 4A), it is more challenging to elucidate, why the candidate cell line CL-40 tested less permissive than CL-14 and CAL-51 despite high *TMPRSS2* transcript levels. CL-40 as well as HCC-1937 hold a missense variation in exon 6 of TMRPSS2 (Fig 4B) causing an amino acid exchange at position 197 (V197M/V160M) within the Scavenger receptor cysteine-rich (SRCR) domain responsible for protein-protein interaction and ligand binding (ExPASy) and is described for various cancer types as well as cell lines (COSMIC). Hence, we speculate that interaction with SARS-CoV-2 might be disturbed by this variant at V197M, which is known for differential allelic frequencies in populations [52].

Importantly, the expression levels of *ACE2*, *TMPRSS2* and *NRP1* are not predictive of SARS-CoV-2 permissiveness per se. While *ACE2* seems to be an essential component, however, it is not sufficient on its own. Permissiveness includes the ability of the virus to enter the cell, replicate, and release infectious progeny. A number of cellular processes can effectively interfere with this replication cycle, including intrinsic immune response, type-I and type-III interferon responses and apoptosis induction [53] and thus determine permissiveness. For SARS-CoV-2, several essential host factors apart from surface receptors were recently discovered [54].

Meanwhile, several organs and cell types have been reported to be infected by SARS-CoV-2, even for those with no or low basal *ACE2* expression [30, 45]. Hence, alternate receptors, proteases and antagonists for SARS-CoV-2 infection could widen the options for viral entry [45] as shown for cathepsin L [55] and CD147 [56]. Besides genetic and epigenetic variations, variable activation of ACE2 could also be triggered by interferon pathways [57, 58].

Seeking for SARS-CoV-2 permissive cell lines by screening *ACE2* RNA-seq-derived expression levels, the human native breast carcinoma cell line CAL-51 and the colon adenocarcinoma cell lines CL-40 and CL-14 have been identified to produce high levels of infectious SARS-CoV-2 virus particles. CAL-51 in particular could enrich SARS-CoV-2 studies trifold: low doubling time of this cell line, viral stock production comparable to commonly used VERO-E6, and a further organ tissue as infection model. Its doubling time is lower than that of the colon carcinoma cell lines CACO-2/CL-14 and the lung carcinoma cell line CALU-3 (Table 1), avoiding the mutation selection pressure to adapt to another species host as seen for the African green monkey cell line VERO-E6 [21–23]. Different replication kinetics as well as different degrees of permissiveness can aid answering specific scientific questions and allow different experimental setups, e.g. in compound screening assays.

In summary, we find that the expression of *ACE2* and *TMPRSS2* are indicative but not sufficient for SARS-CoV-2 productive infection in human cell lines, probably partly due to a truncated *ACE2* splice variant and *TMPRSS2* variants. Out of 29 tested cancer cell lines, we have identified three highly and 11 lowly permissive cell lines that vastly broaden the choice for SARS-CoV-2 cell culture models. The breast carcinoma cell line CAL-51 in particular may serve as a suitable cell line for SARS-CoV-2 studies for viral propagation because of its easy management.

## Methods

### CCLE RNA-seq data set analysis

RNA-seq data were retrieved and pre-processed as described [59]. Briefly, alignment files were downloaded from CGHub via genetorrent, sorted (samtools 0.1.19) and converted to fastq files (bedtools v2.21.0). Reads were trimmed via fastq-mcf (ea-utils 1.1.2–686). STAR (2.5.3a) served as read mapper to the human genome Gencode 26, HTSeq (0.11.3) was used as count tool. Raw counts were normalised and transformed to FPKM (fragments per kilobase million) via R/Bioconductor (3.6.9) loading DESeq2 (1.26.0) and visualised via ggplot2 (3.1.1). Different exon usage was discovered by visualising the alignment files via IGV (2.8.0) [60]. For detecting mutations, mapped reads were assigned to read groups (picard, 2.9.2 [www.broadinstitute.github.io/picard]); reads were split, trimmed and reassigned (GenomeAnalysisTK, 3.7–0, SplitNCigarReads [61]); mutations were called by the HaplotypeCaller (GenomeAnalysisTK, 3.7–0,) [61]; and mutation effects were predicted via the Ensembl VEP (GRCh38, v90) [62]. Mutations were filtered to ≥10 depth.

### Cultivation of human cell lines

The continuous cell lines are part of the cell culture collection at DSMZ (https://www.dsmz.de/collection/catalogue/human-and-animal-cell-lines). Tissue origin, doubling-time, growth properties, cytogenetics and more information on these cell lines can be retrieved from the DSMZ website. Cell lines were grown at 37°C in a humidified atmosphere of air containing 5% CO2. The basic growth media (Life Technologies, Darmstadt, Germany) were supplemented with 10–20% fetal bovine serum (Sigma Aldrich, Taufkirchen, Germany). No antibiotics were added to the cultures. All cell lines were free of mycoplasma and other bacterial, yeast and fungi contaminations as tested by PCR and microbiological growth assays [63]. The authenticity of the cell lines was determined by DNA typing [64]. The cells were first cultured in cell culture flasks to confluence, detached with trypsin/EDTA, washed with PBS and pelleted for RNA and protein preparation. For virus infection experiments the cells were seeded in 96-well plates at different densities starting with 0.25—1.5 x 105 cells per well as highest cell number depending on the cell line. Optimal cell densities were determined by observing the growth progression with an IncuCyte instrument (Essen Bioscience, Hertfordshire, UK). Cells were then seeded at semi-confluence or complete confluence and three further dilutions each with half of the previous cell densities. The cells were grown for one day before the virus infection experiments were started.

### qRT-PCR analysis

Quantitative Real-time polymerase chain reaction (qRT-PCR) analysis for human mRNA was performed to quantify specific transcripts in the cells. Total RNA was extracted from cell line samples using TRIzol reagent (Fisher Scientific, Schwerte, Germany). cDNA was generated from 1 μg RNA by random priming using the Biozym cDNA Synthesis Kit (Biozym, Hessisch Oldendorf, Germany). qRT-PCR analysis was performed with the 7500 Real-time System, using commercial buffer and primer sets for the amplification of *ACE2* (Hs01085333_m1) and *TMPRSS2* (Hs01122322_m1) transcripts (Thermo Fisher Scientific, Darmstadt, Germany).

Viral RNA of infected cell cultures was extracted using spin filter membranes (innuPREP Virus TS RNA Kit from Analytik Jena) according to the manufacturer’s recommendations. Briefly, cells were lysed in the presence of carrier RNA and Proteinase K at 70°C for 10 min. Nucleic acids were then bound to the surface of the spin filter membranes, washed and subsequently eluted with RNase-free water. One to five *μ*g RNA were then reverse transcribed to cDNA applying random hexamers. The primers for the qRT-PCR for the amplification of the SARS-CoV-2 N gene were N_Sarbeco_F (5´-CACATTGGCACCCGCAATC-3’) and N_Sarbeco_R (5’-GAGGAACGAGAAGAGGCTTG-3’) [65].

We analysed the transcript of TATA box binding protein (TBP) for normalisation of expression levels using SYBR Green for quantification. Quantitative analyses were performed in triplicate, if not indicated differently, analysed and visualised with R/Bioconductor packages ddCt and ggplot2.

### Western blots

Antibodies against ACE2 (ab15348), TMPRSS2 (ab109131), and GAPDH (ab8245) were obtained from Abcam (Cambridge, UK). Western blot samples were prepared as described previously [66]. Proteins on nitrocellulose membranes were visualized with the biotin/streptavidin-horseradish peroxidase system (GE Healthcare; Little Chalfont, UK) in combination with the “Renaissance Western Blot Chemoluminescence Reagent” (Perkin Elmer; Waltham, MA, USA). Documentation was performed using the digital system ChemoStar Imager (INTAS, Göttingen, Germany).

### Viral infection of cell lines

Infection with SARS-CoV-2 was performed with virus strain Zagreb (SARS-CoV2/ZG/297–20, University Hospital for Infectious Diseases, Zagreb, Croatia). If not indicated otherwise, cells were seeded onto 96-well cell culture plates and infected 24–48 hours later with 250 pfu/well using a 10 *μ*l inoculum and 100 *μ*l total well volume.

### Virus plaque and neutralisation assays

Quantification of SARS-CoV-2 infectious units was done via a virus plaque assay. Supernatants of infected cells were taken 1–4 dpi, serially diluted, and used to infect confluent VERO-E6 cells on 96-well cell culture plates for one hour. Then, the inoculum was removed and cells were overlaid with cell culture medium (MEM, 10% FCS, 2 mM glutamine) containing 1.5% methyl-cellulose. After 3 days, virus plaques were counted from phase contrast microscopic images taken with a Sartorius IncuCyte S3 at 4x magnification.

For neutralisation assays antibody COR-101 and virus were mixed and incubated for 1h at 37°C and applied to cells for 24 hpi. COR-101 for was a kind gift of Corat Therapeutics GmbH (Braunschweig, Germany).

## Supporting information

S1 Fig Original western blots. Original western blots to cropped western blot images in Fig 2B.
(PDF)

S2 Fig Virus titers. Virus titers in the supernatant of infected cell lines at 1-4 dpi determined by plaque assay for the top 11-25 *ACE2* expressing, two neuronal (DBTRG-05MG, KELLY), and two oral squamous cell carcinoma (UPCI-SCC-074, UPCI-SCC-131) cell lines. The red dotted line indicates the initial inoculum (2000 pfu/ml); the blue dashed line specifies the minimal limit of detection LOD 33.3 pfu/ml. Number of replicates varied between 3-8. pfu: plaque-forming units; mean values with SEM.
(TIFF)

S3 Fig Neutralisation assays. Neutralising SARS-CoV-2 antibody was applied to the high permissive cell lines CAL-51 and CL-14 and the control cell line VERO-E6. With increasing antibody concentration viral production is decreasing in all three cell lines. The red dotted line indicates the initial inoculum (2000 pfu/ml); the blue dashed line specifies the minimal limit of detection LOD 33.3 pfu/ml. Note the logarithmic scaling for the x-axis.
(TIF)

S4 Fig *ACE2* splicing variant. Different exon usage of *ACE2* for the top 25 *ACE2* expressing cell lines plus CALU-3. RNA-seq data were visualised via IGV and cell lines ordered alphabetically.
(TIFF)

S1 Table Summary cell lines. Gene expression, splice and single nucleotide variants of *ACE2* and *TMPRSS2* in the cell lines used in this study. These were identified on the basis of CCLE RNA-seq data (FPKM, see Material and methods). +truncated splice variant observed; 0/1 heterozygous, 1/1 homozygous mutation.
(ODS)
