# Supplementary figures and images for: Identification of cell lines CL-14, CL-40 and CAL-51 as suitable models for SARS-CoV-2 infection studies
Source: PLoS One. 2021 Aug 2;16(8):e0255622. doi: 10.1371/journal.pone.0255622 (PMC8328321; doi:10.1371/journal.pone.0255622)

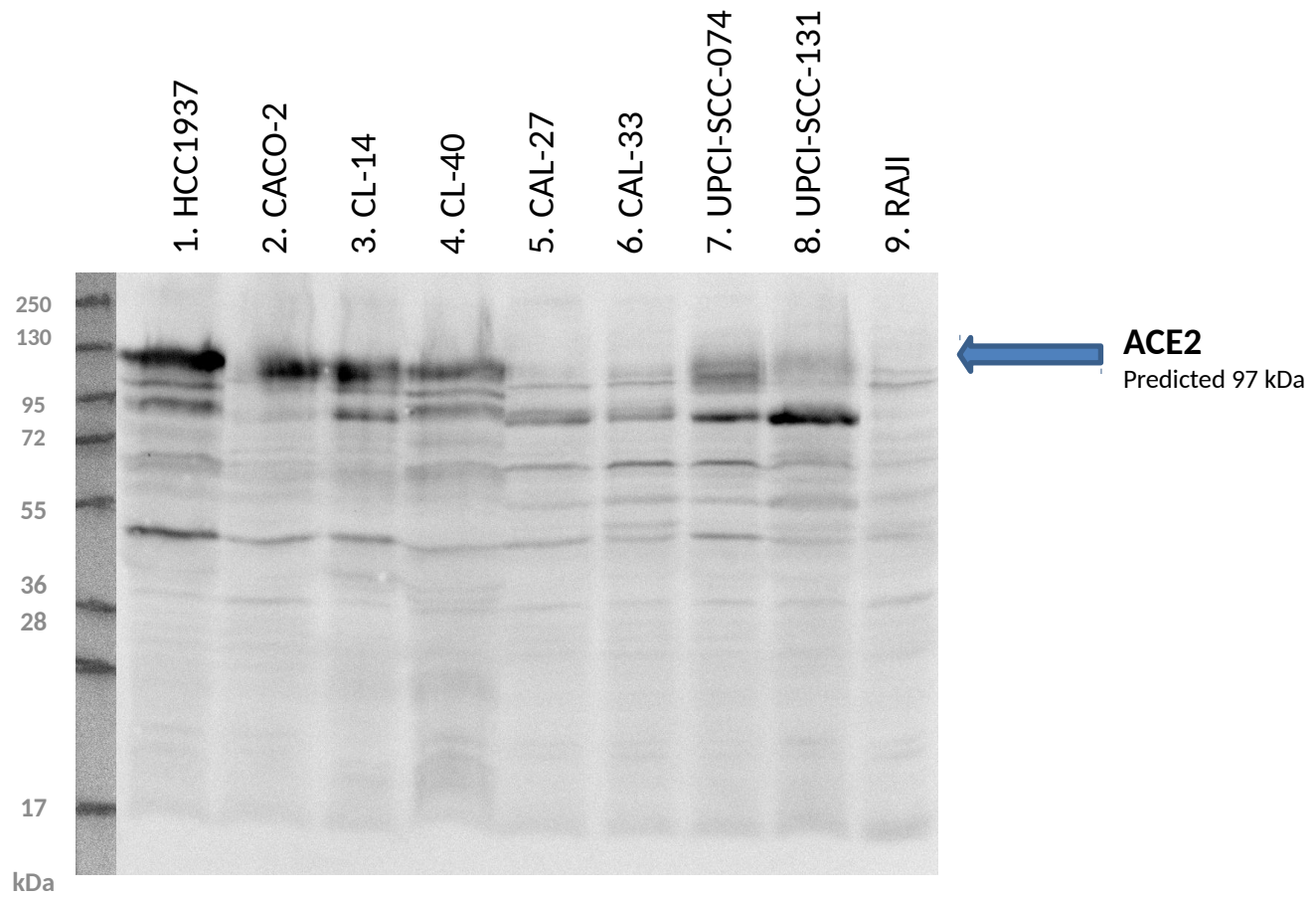

**Anti ACE2**

Abcam ab 15348

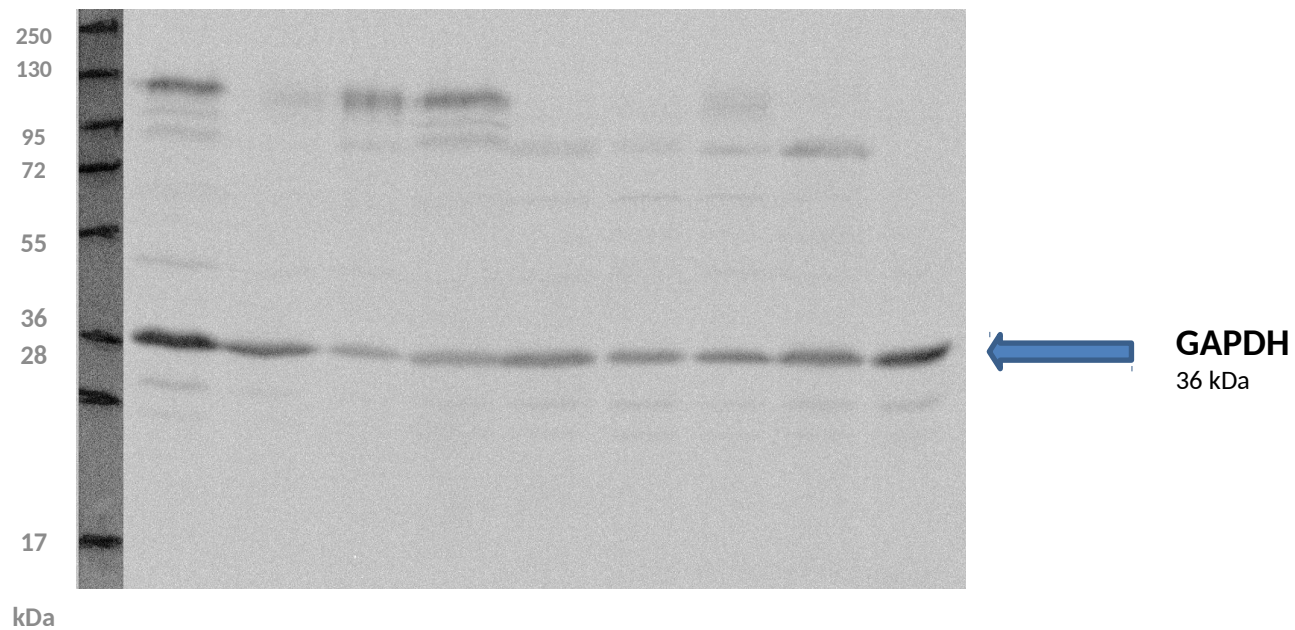

For ACE2

Anti GAPDH

Abcam ab 8245

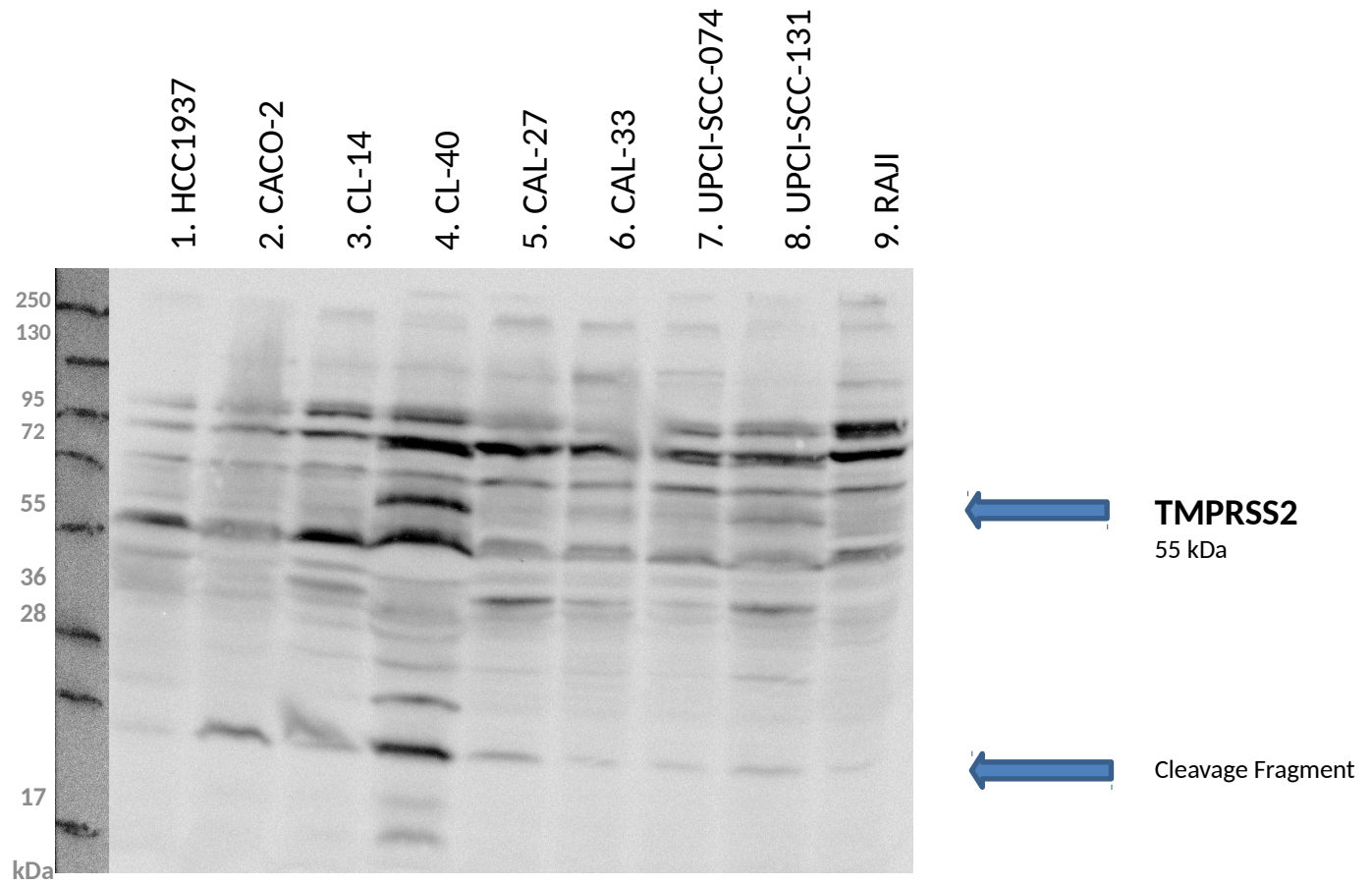

**Anti TMPRSS2**

Abcam ab 109131

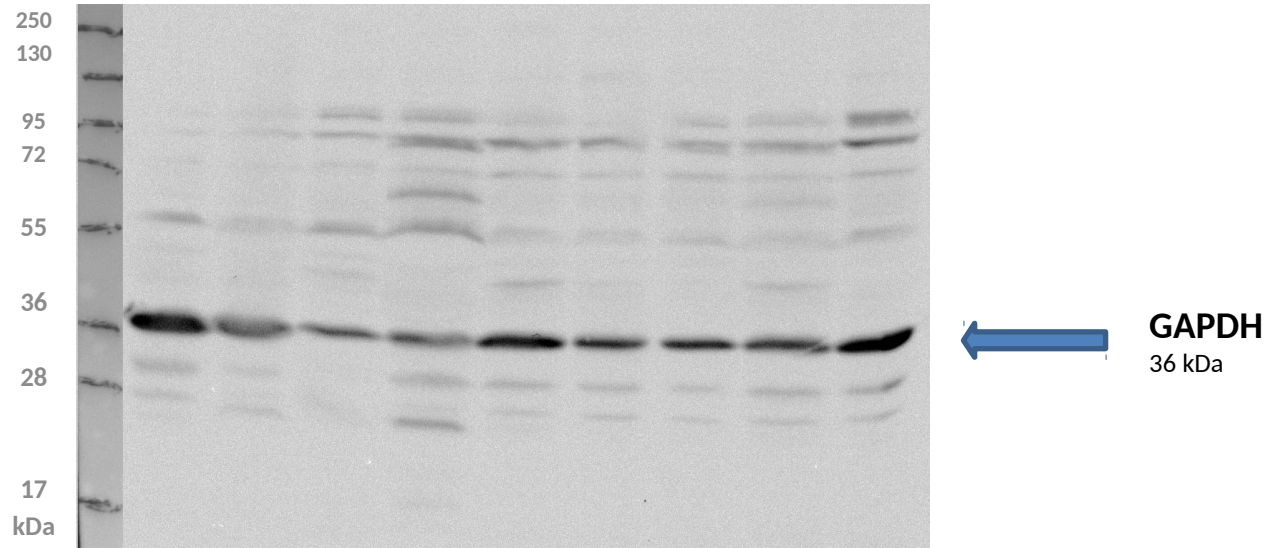

For TMPRSS2

Anti GAPDH

Abcam ab 8245

Supplement: S1 Fig — Original western blots to cropped western blot images in Fig 2B. (PDF) [file pone.0255622.s001.pdf]

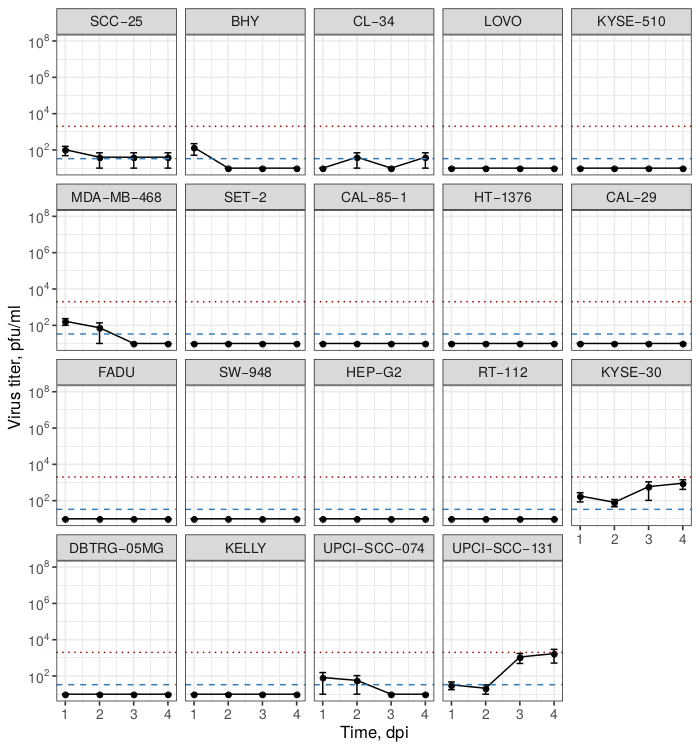

Supplement: S2 Fig — Virus titers in the supernatant of infected cell lines at 1-4 dpi determined by plaque assay for the top 11-25 ACE2 expressing, two neuronal (DBTRG-05MG, KELLY), and two oral squamous cell carcinoma (UPCI-SCC-074, UPCI-SCC-131) cell lines. The red dotted line indicates the initial inoculum (2000 pfu/ml); the blue dashed line specifies the minimal limit of detection LOD 33.3 pfu/ml. Number of replicates varied between 3-8. pfu: plaque-forming units; mean values with SEM. (TIFF) [file pone.0255622.s002.tiff]

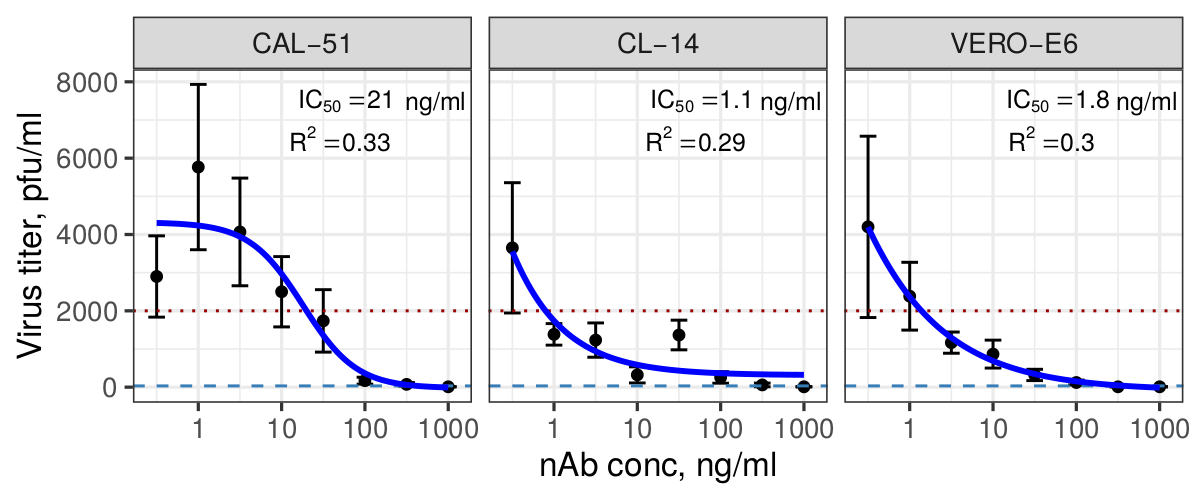

Supplement: S3 Fig — Neutralising SARS-CoV-2 antibody was applied to the high permissive cell lines CAL-51 and CL-14 and the control cell line VERO-E6. With increasing antibody concentration viral production is decreasing in all three cell lines. The red dotted line indicates the initial inoculum (2000 pfu/ml); the blue dashed line specifies the minimal limit of detection LOD 33.3 pfu/ml. Note the logarithmic scaling for the x-axis. (TIF) [file pone.0255622.s003.tif]

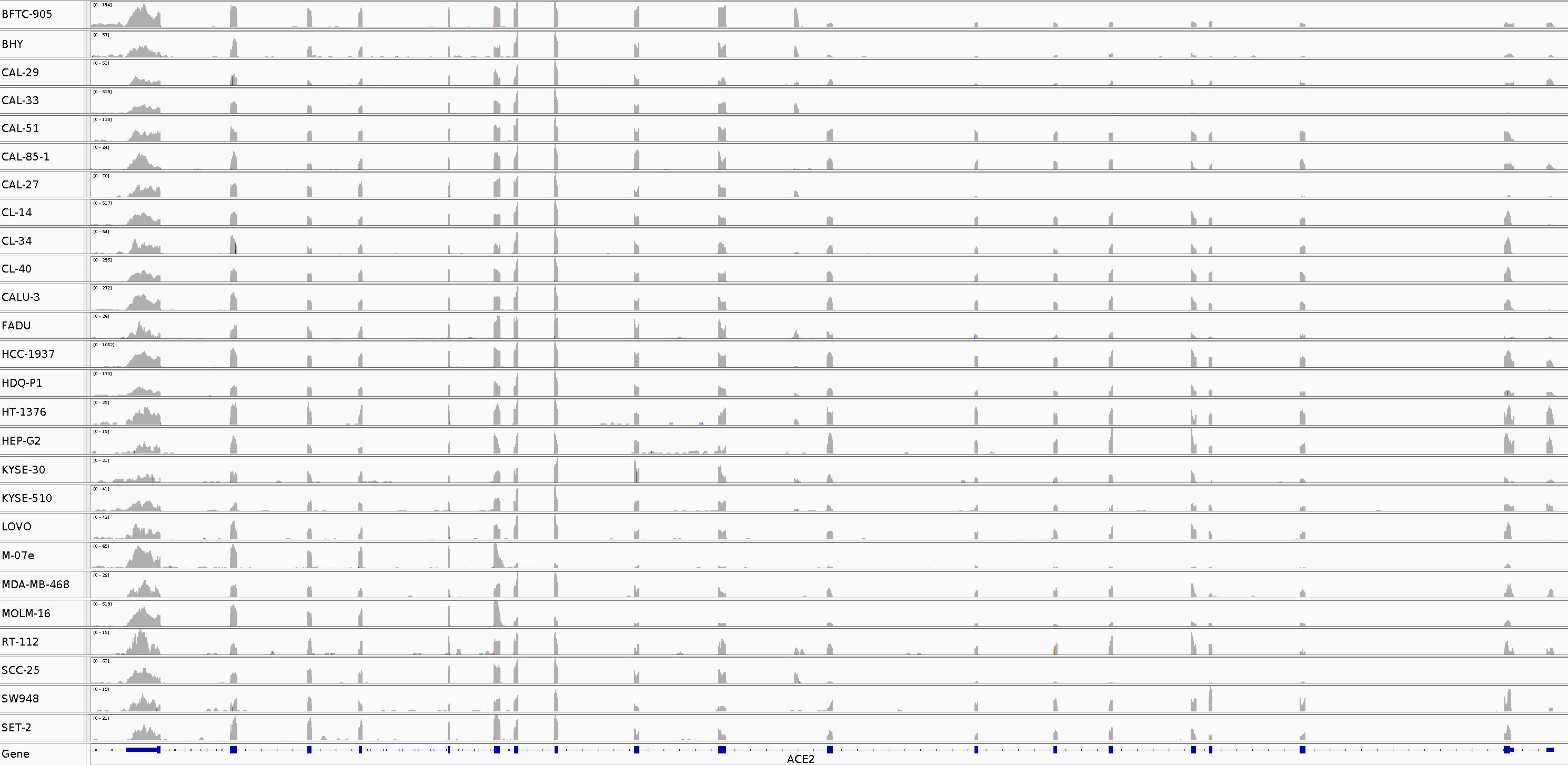

Supplement: S4 Fig — Different exon usage of ACE2 for the top 25 ACE2 expressing cell lines plus CALU-3. RNA-seq data were visualised via IGV and cell lines ordered alphabetically. (TIFF) [file pone.0255622.s004.tiff]
